# Unexpected Routes of the Mutagenic Tautomerization of the T Nucleobase in the Classical A·T DNA Base Pairs: A QM/QTAIM Comprehensive View

**DOI:** 10.3389/fchem.2018.00532

**Published:** 2018-11-27

**Authors:** Ol'ha O. Brovarets', Kostiantyn S. Tsiupa, Andrii Dinets, Dmytro M. Hovorun

**Affiliations:** ^1^Department of Molecular and Quantum Biophysics, Institute of Molecular Biology and Genetics, National Academy of Sciences of Ukraine, Kyiv, Ukraine; ^2^Department of Pharmacology, Bogomolets National Medical University, Kyiv, Ukraine; ^3^Department of Surgery #4, Bogomolets National Medical University, Kyiv, Ukraine; ^4^Department of Pathophysiology, Bogomolets National Medical University, Kyiv, Ukraine

**Keywords:** mutagenic tautomerisation, transition state, proton transfer, Watson-Crick, reverse Watson-Crick, Hoogsteen and reverse Hoogsteen, classical A·T DNA base pairs, wobble structure

## Abstract

In this paper using quantum-mechanical (QM) calculations in combination with Bader's quantum theory of “Atoms in Molecules” (QTAIM) in the continuum with ε = 1, we have theoretically demonstrated for the first time that revealed recently highly-energetic conformers of the classical A·T DNA base pairs – Watson-Crick [A·T(w_WC_)], reverse Watson-Crick [A·T(w_rWC_)], Hoogsteen [A·T(w_H_)] and reverse Hoogsteen [A·T(w_rH_)] – act as intermediates of the intrapair mutagenic tautomerization of the T nucleobase owing to the novel tautomerisation pathways: A·T(w_WC_)↔A·T^*^(w^⊥^_WC_); A·T(w_rWC_)↔A·${\text{T}}_{\text{O}2}^{*}$(w^⊥^_rWC_); A·T(w_H_)↔A·T^*^(w^⊥^_H_); A·T(w_rH_)↔A·${\text{T}}_{\text{O}2}^{*}$(w^⊥^_rH_). All of them occur *via* the transition states as tight ion pairs (A^+^, protonated by the N6H_2_ amino group)·(T^−^, deprotonated by the N3H group) with quasi-orthogonal geometry, which are stabilized by the participation of the strong (A)N6^+^H···O4^−^/O2^−^(T) and (A)N6^+^H···N3^−^(T) H-bonds. Established tautomerizations proceed through a two-step mechanism of the protons moving in the opposite directions along the intermolecular H-bonds. Initially, proton moves from the N3H imino group of T to the N6H_2_ amino group of A and then subsequently from the protonated N6^+^H_3_ amino group of A to the O4/O2 oxygen atom of T, leading to the products – A·T^*^(w^⊥^_WC_), A·${\text{T}}_{\text{O}2}^{*}$(w^⊥^_rWC_), A·T^*^(w^⊥^_H_), and A·${\text{T}}_{\text{O}2}^{*}$(w^⊥^_rH_), which are substantially non-planar, conformationally-labile complexes. These mispairs are stabilized by the participation of the (A)N6H/N6H'···N3(T) and (T)O2H/O4H···N6(A) H-bonds, for which the pyramidalized amino group of A is their donor and acceptor. The Gibbs free energy of activation of these mutagenic tautomerizations lies in the range of 27.8–29.8 kcal·mol^−1^ at *T* = 298.15 K in the continuum with ε = 1.

## Introduction

Clarification at the microstructural level of the physico-chemical mechanisms underlying the formation of the mutagenic tautomers of the DNA bases *via* the mutagenic tautomerization of the classical Watson-Crick DNA base pairs is a matter of extreme importance for such branches of life science as molecular biophysics and molecular biology, since it enables us to understand the sources of the genome instability (Watson and Crick, 1953a,b; Löwdin, 1963, 1966; Topal and Fresco, 1976). Genome instability is frequently associated with mutations in DNA, playing role in cancer development due to DNA replication errors (Liu et al., 2014; Tomasetti et al., 2017).

Mutagenic tautomerization of the DNA bases attracts researchers' curiosity since the establishment of the spatial architecture of DNA molecule (Watson and Crick, 1953a) and further formulation of the tautomeric hypothesis of the origin of the spontaneous point mutations by Watson and Crick (Watson and Crick, 1953b).

Distinguished quantum chemist Per-Orlov Löwdin proposed original idea based on the electronic structure of the complementary A·T and G·C pairs of the DNA bases (Löwdin, 1963, 1966), which makes possible their conversion into the high-energy tautomerized states – A^*^·T^*^(L) and G^*^·C^*^(L) base pairs [currently known as Löwdin's base pairs; here and below rare, in particular mutagenic (Brovarets' and Hovorun, 2010a; Brovarets', 2015), tautomers are marked with an asterisk] causing origin of the transitions and transversions during the DNA replication. Löwdin believed that these transformations should be carried out by the double proton transfer (DPT) in the opposite directions along the neighboring intermolecular hydrogen (H) bonds through the quantum tunneling. These representations played an extremely important role in the formation of new visions in quantum biology and attracted the attention of a wide range of Löwdin's followers (Florian et al., 1994; Gorb et al., 2004; Bertran et al., 2006; Cerón-Carrasco and Jacquemin, 2013; Maximoff et al., 2017).

However, from the physico-chemical point of view it was established that generally accepted Löwdin's mechanism of the DPT along the intermolecular H-bonds in the Watson-Crick DNA base pairs cannot be the source of the formation of the mutagenic tautomers of the nucleobases due to the absence of the reverse barrier of tautomerization in the A·T(WC) pair of the DNA bases and its small value in comparison with *kT* (0.62 kcal·mol^−1^ at *T* = 298.15 K) for the G·C(WC) DNA base pair (Gorb et al., 2004; Bertran et al., 2006; Brovarets' et al., 2012; Brovarets' and Hovorun, 2014a,b, 2015a).

Recently, we have proposed another mechanism of the mutagenic tautomerization of the A·T(WC) and G·C(WC) pairs of the DNA bases, which is alternative to Löwdin's approach, occurring *via* the sequential intrapair proton transfer and shifting of the bases relative each other, which ultimately leads to the wobble configuration (Brovarets' and Hovorun, 2015b). Moreover, we have discovered this intrinsic ability to perform wobble↔Watson-Crick / Watson-Crick↔wobble tautomeric transitions *via* the sequential intrapair proton transfer for all possible incorrect base mispairs, which are active players in the field of the spontaneous point mutagenesis: purine·pyrimidine – G·T and A·C (Brovarets' and Hovorun, 2009, 2015c,d, 2016), purine·purine – A·A, A·G and G·G (Brovarets' and Hovorun, 2015e,f) and pyrimidine·pyrimidine – C·C, C·T and T·T (Brovarets' and Hovorun, 2015f,g). Notably, these interconverisons are accompanied by a significant rebuilding of the base mispairs with Watson-Crick architecture into the mismatches wobbled toward both minor and major DNA grooves and *vice versa*. Moreover, it was established that these tautomerisation reactions occur non-dissociatively and are accompanied by the consequent replacement of the unique patterns of the intermolecular specific interactions along intrinsic reaction coordinate (IRC) (Brovarets' et al., 2013, 2017a,b).

These data allows to suggest that the intrapair tautomeric transition of the wobble pairs from the main tautomeric form into the rare, mutagenic, having a WC or close to its configuration, and *vice versa*, is the key to understanding of the microstructural mechanisms of the emergence of the spontaneous transitions and transversions at the DNA replication (Brovarets' and Hovorun, 2009, 2015b,c,d, 2016). Moreover, these theoretical approaches have been partly experimentally confirmed for some DNA/RNA purine·pyrimidine pairs (Nedderman et al., 1991, 1993; Kimsey et al., 2015, 2018).

**Scheme 1:**
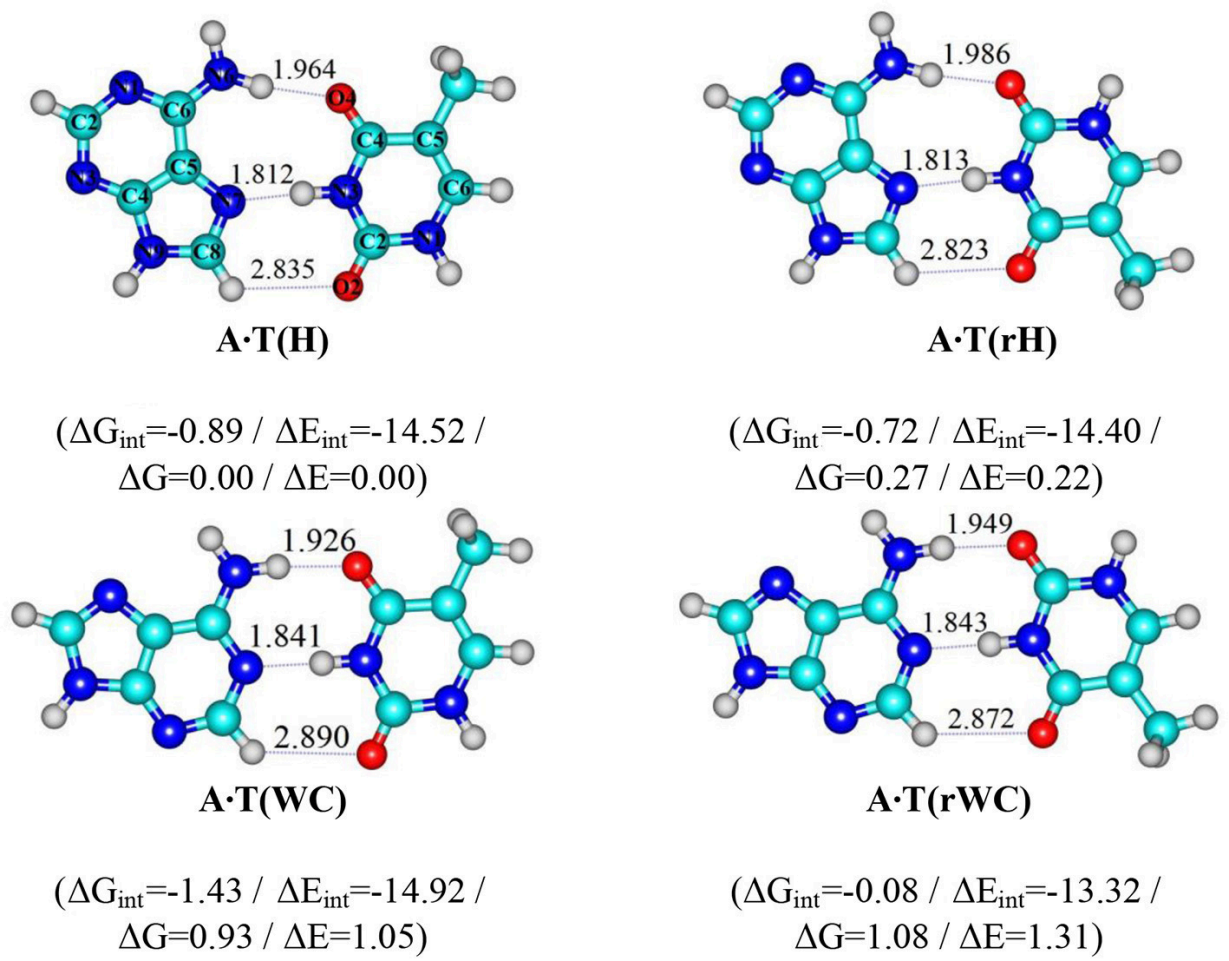
Geometrical structures of the classical A·T DNA base pairs – Hoogsteen A·T(H), reverse Hoogsteen A·T(rH), Watson-Crick A·T(WC), reverse Watson-Crick A·T(rWC) (Brovarets', 2013b). Electronic ΔE_int_ and Gibbs free ΔG_int_ energies of the interaction (MP2/6-311++G(2df,pd)//B3LYP/6-311++G(d,p) level of theory, in kcal·mol-1), relative Gibbs free energies ΔG and electronic energies ΔE (MP2/aug-cc-pVDZ//B3LYP/6-311++G(d,p) level of theory in the continuum with ε = 1 at T = 298.15 *K* in kcal·mol-1) are presented below complexes in brackets. Dotted lines indicate AH··· B H-bonds – their lengths H··· B are presented in angstroms; carbon atoms are in light-blue, nitrogen – in dark-blue, hydrogen – in grey and oxygen – in red.

In this study, we succeeded to further elaborate such approach and to reveal new mechanism of the mutagenic tautomerization of the classical A·T DNA base pairs (Scheme 1) as their intrinsic property, lying beyond classical representations at the microstructural level and which was not presented in the literature before. For the first time, it was theoretically shown using QM/QTAIM methods, that the transition of these pairs into the substantially non-planar, high-energy conformers (Brovarets' et al., 2018a) provokes intrapair mutagenic tautomerization of the T DNA base from the canonical, diketo into the rare, enol tautomeric forms T^*^ and ${\text{T}}_{\text{O}2}^{*}$ (Brovarets' and Hovorun, 2014a, 2015b,d; Brovarets' et al., 2014a, 2015). Moreover, for the first time we have investigated in details conformationally-tautomeric properties of the classical A·T DNA base pairs (Brovarets' et al., 2018b,c,d,e).

Transition states (TSs) of these mutagenic tautomerisations are tight ion pairs (A^+^, protonated by the N6H_2_ amino group; T^−^, deprotonated by the N3H group) with quasi-orthogonal geometry, which are stabilized by the participation of the strong (A)N6^+^H··· O4^−^/O2^−^(T) and (A)N6^+^H··· N3^−^(T) H-bonds. Discovered reaction of the mutagenic tautomerization proceeds through the stepwise mechanism of the PT along the H-bonds: primarily proton moves from the imino group N3H of T to the N6H_2_ amino group of A and then proton transfers from the protonated N6^+^H_3_ amino group of A to the O4/O2 oxygen atom of T, leading to the products, which are substantially non-planar, conformationally-labile complexes. These complexes are stabilized by the participation of the (A)N6H/N6H′··· N3(T) and (T)O2H/O4H··· N6(A) H-bonds, for which the pyramidalized amino group of A DNA base acts as their donor and acceptor. The Gibbs free energy of the activation of the mutagenic tautomerizations lies in the range of 27.79–29.83 kcal·mol^−1^ at *T* = 298.15 K in the continuum with ε = 1.

Also in this study, it was shown that the formed A·T^*^(w^⊥^_WC_), A·T^*^(w^⊥^_H_), A·${\text{T}}_{\text{O}2}^{*}$(w^⊥^_rWC_) and A·${\text{T}}_{\text{O}2}^{*}$(w^⊥^_rH_) complexes can conformationally interconvert according to the pathways A·T^*^(w^⊥^_WC_)↔A·T^*^(w^⊥^_H_) and A·${\text{T}}_{\text{O}2}^{*}$(w^⊥^_rWC_)↔A·${\text{T}}_{\text{O}2}^{*}$(w^⊥^_rH_) through three different TSs.

## Computational methods

Geometries of the investigated DNA base pairs and TSs of their mutual tautomeric and conformational transformations, as well as their harmonic vibrational frequencies were calculated at the B3LYP/6-311++G(d,p) level of theory (Hariharan and Pople, 1973; Krishnan et al., 1980; Lee et al., 1988; Parr and Yang, 1989; Tirado-Rives and Jorgensen, 2008), using Gaussian'09 package (Frisch et al., 2009) followed by the IRC calculations in the forward and reverse directions from each TS using Hessian-based predictor-corrector integration algorithm (Hratchian and Schlegel, 2005). A scaling factor that is equal to 0.9668 (Brovarets' and Hovorun, 2010b,c,d, 2011; El-Sayed et al., 2015) was applied in this study for the correction of the harmonic frequencies of all DNA base pairs and TSs of their tautomeric and conformational transitions. We have confirmed the TSs, localized by Synchronous Transit-guided Quasi-Newton method (Peng et al., 1996), on the potential energy landscape by the presence of one and only one imaginary frequency in the vibrational spectra of the complexes. We applied standard TS theory for the estimation of the activation barriers of the tautomeric transformations (Atkins, 1998). Single point electronic energy calculations have been performed using MP2 level of theory (Frisch et al., 1990) and aug-cc-pVDZ Dunning's cc-type basis set (Kendall et al., 1992), which was confirmed as appropriate level of theory for the analogous systems and tasks (Lozynski et al., 1998; Danilov et al., 2005; Matta, 2010; Rutledge and Wetmore, 2012; Brovarets' and Pérez-Sánchez, 2016, 2017; Brovarets' et al., 2016, 2018f; Brovarets' and Hovorun, 2018a).

All calculations were performed for the base pairs in the continuum with a dielectric constant of ε = 1 as their intrinsic property, that is adequate for modeling of the processes occurring in real systems (Bayley, 1951; Dewar and Storch, 1985; Petrushka et al., 1986; García-Moreno et al., 1997; Mertz and Krishtalik, 2000; Bebenek et al., 2011; Wang et al., 2011; Maximoff et al., 2017) without deprivation of the structurally functional properties of the bases in the composition of DNA (Brovarets' and Pérez-Sánchez, 2016, 2017; Brovarets' et al., 2016, 2018f).

The Gibbs free energy G for all structures was obtained in the following way:

(1) $$\begin{array}{l} \text{G}={\text{E}}_{\text{el}}+{\text{E}}_{\text{corr}}, \end{array}$$

where E_el_ - electronic energy, while E_corr_ - thermal correction.

The Gibbs free energy of activation or barrier for the forward tautomeric/conformational transition was calculated as the difference between the Gibbs free energy of the TS and reactant of the reaction. The Gibbs free energy for the reverse tautomeric/conformational transition was calculated as the difference between the Gibbs free energy of the TS and product of the reaction.

Electronic interaction energies ΔE_int_ were calculated at the MP2/6-311++G(2df,pd) level of theory as the difference between the total energy of the base pair and energies of the monomers and corrected for the basis set superposition error (BSSE) (Boys and Bernardi, 1970; Gutowski et al., 1986) through the counterpoise procedure (Sordo et al., 1988; Sordo, 2001).

Bader's quantum theory of Atoms in Molecules (QTAIM) (Bader, 1990; Matta and Hernández-Trujillo, 2003; Matta, 2014; Lecomte et al., 2015) was applied to analyse the electron density distribution, using software package AIMAll (Keith, 2010). The presence of the bond critical point (BCP), namely (3,−1) BCP, and a bond path between hydrogen donor and acceptor or between two electronegative covalently bonded atoms, as well as the positive value of the Laplacian at this BCP (Δρ> 0), were considered as criteria for the H-bond or attractive van der Waals contact formation (Matta et al., 2006; Brovarets' and Hovorun, 2014c, 2018b; Brovarets' et al., 2014b). Wave functions were obtained at the level of theory used for geometry optimisation.

The energies of the attractive van der Waals contacts (Matta and Boyd, 2007; Brovarets' et al., 2018a) in the TSs of the conformational transitions of the tautomerized base pairs were calculated by the empirical Espinosa-Molins-Lecomte (EML) formula (Espinosa et al., 1998; Mata et al., 2011), based on the electron density distribution at the (3,−1) BCPs of the specific contacts:

(2) $$\begin{array}{l} E=0.5\cdot \text{V}\left(\text{r}\right), \end{array}$$

in this formula V(r) is a value of a local potential energy at the (3,−1) BCP.

The energies of the conventional AH···B H-bonds were evaluated by the empirical Iogansen's formula (Iogansen, 1999):

(3) $$\begin{array}{l} E_{AH\cdot \cdot \cdot B}=0.33\cdot \sqrt{△v-40}, \end{array}$$

in this formula Δν is a magnitude of the frequency shift of the stretching mode of the AH H-bonded group involved in the AH···B H-bond relatively the unbound group. The partial deuteration was applied in order to avoid the effect of vibrational resonances (Brovarets' and Hovorun, 2015h; Brovarets' et al., 2018a).

The atomic numbering scheme for the DNA bases was conventional (Saenger, 1984).

## Obtained results and discussion

**Figure 1:**
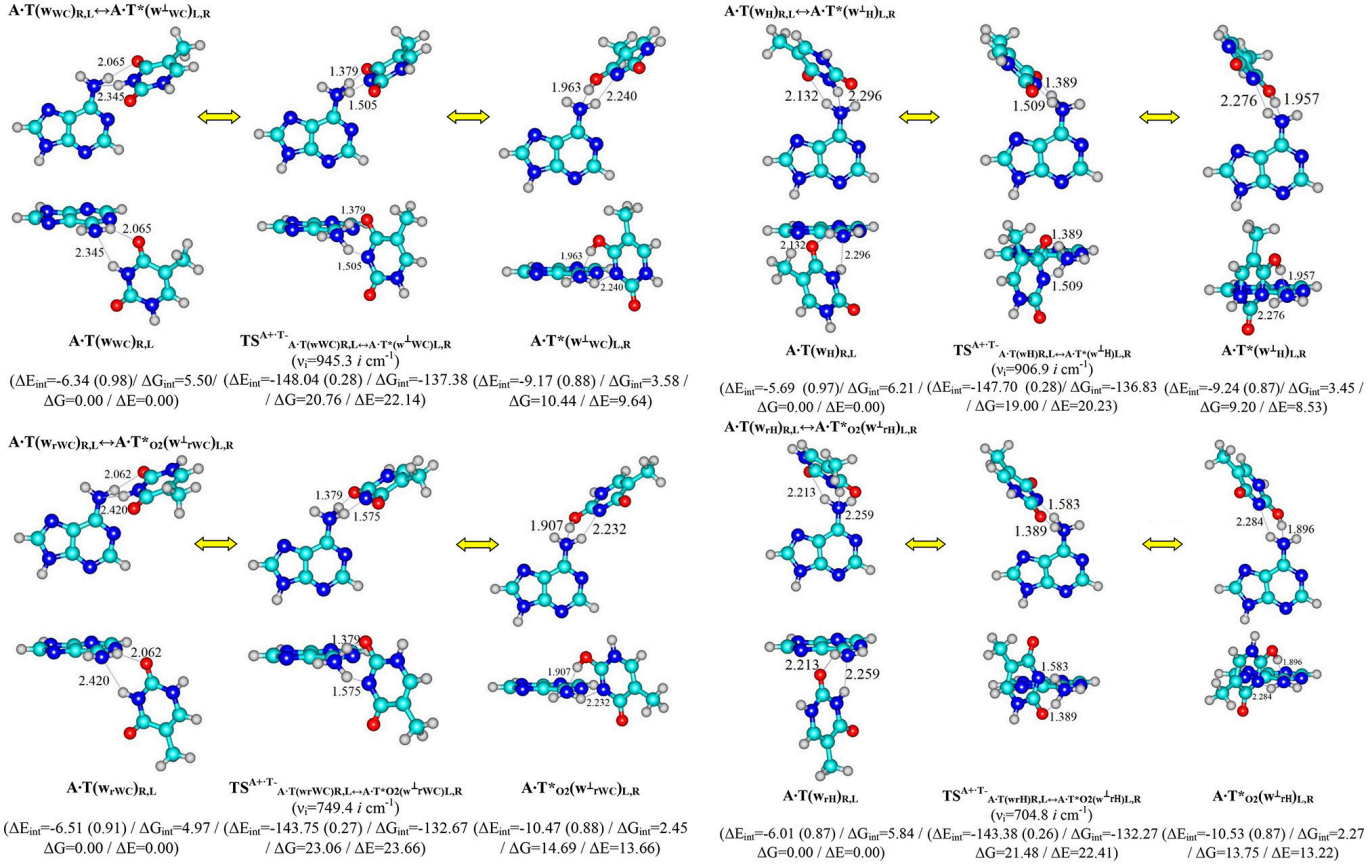
Discovered new reaction pathways of the T mutagenic tautomerization in the classical A·T DNA base pairs through the double proton transfer. νi – imaginary frequencies at the TSs of the conformational transitions. For more designations refer to Scheme 1.

In our previous study, for the first time we have succeeded to establish in the classical biologically-important A·T DNA base pairs with C_s_ symmetry – Watson-Crick (WC), reverse Watson-Crick A·T(rWC), Hoogsteen A·T(H) and reverse Hoogsteen A·T(rH) DNA base pairs (Scheme 1) (Donohue and Trueblood, 1960; Haschemeyer and Sobell, 1963; Hoogsteen, 1963; Brovarets', 2013a,b; Yang et al., 2015; Poltev et al., 2016; Zhou, 2016; Szabat and Kierzek, 2017) – novel high-energetic, dynamically-stable, mirror-symmetrical A·T(w_WC_)_R, L_, A·T(w_H_)_R, L_, A·T(w_rWC_)_R, L_ and A·T(w_rH_)_R, L_ conformational states (Figure 1) (Brovarets' et al., 2018a). Their distinguished feature is significantly non-planar structure (C_1_ symmetry), which is caused by the pyramidal structure of the ≥C6N6H_2_ amino fragment of the A DNA base, which amino group acts simultaneously as a donor and an acceptor of the specific intermolecular interactions with T DNA base by two (T)N3H··· N6(A) and (A)N6H/N6H′··· O4/O2(T) H-bonds (the N6H′ bond has *trans-*orientation relatively the N1C6 bond of A). Each of the four A·T Watson-Crick DNA base pairs transfers into the aforementioned conformers *via* two mirror-symmetric pathways through the TS_A·T(WC)↔A·T(_w__WC_)R, L_, TS_A·T(rWC)↔A·T(_w__rWC_)R, L_, TS_A·T(H)↔A·T(_w__H_)R, L_ and TS_A·T(rH)↔A·T(_w__rH_)R, L_ (C_1_ symmetry). At this, mirror-symmetrical complexes, which are enantiomers, are marked with the subscripts R and L. Notably, enantiomers in the achiral environment demonstrate identical scalar physico-chemical characteristics and differ only by the direction of the dipole moment.

Possible biological role of these conformers was also elucidated, in particular – their participation in the non-dissociative conformational interconversions of all four classical A·T DNA base pairs (Brovarets' et al., 2018b,e). Recently, we have identified novel pathway of the mutagenic tautomerisation of these structures through the quasi-orthogonal transition state as A^−^·T^+^ (Brovarets' et al., 2018c).

These data inspired us to elaborate further this novel point of view for the classical objects such as biologically-important A·T DNA base pairs and allow to suggest the possibility of the mutagenic tautomerization of T through the stepwise PT along the appropriate intermolecular H-bonds from the N3H imino group of T to the N6 atom of the N6H_2_ amino group of A in the just-mentioned conformers and further – from the protonated amino group ${\text{NH}}_{3}^{+}$ of A to the O4/O2 oxygen atoms of T depending on the starting pair.

**Figure 2:**
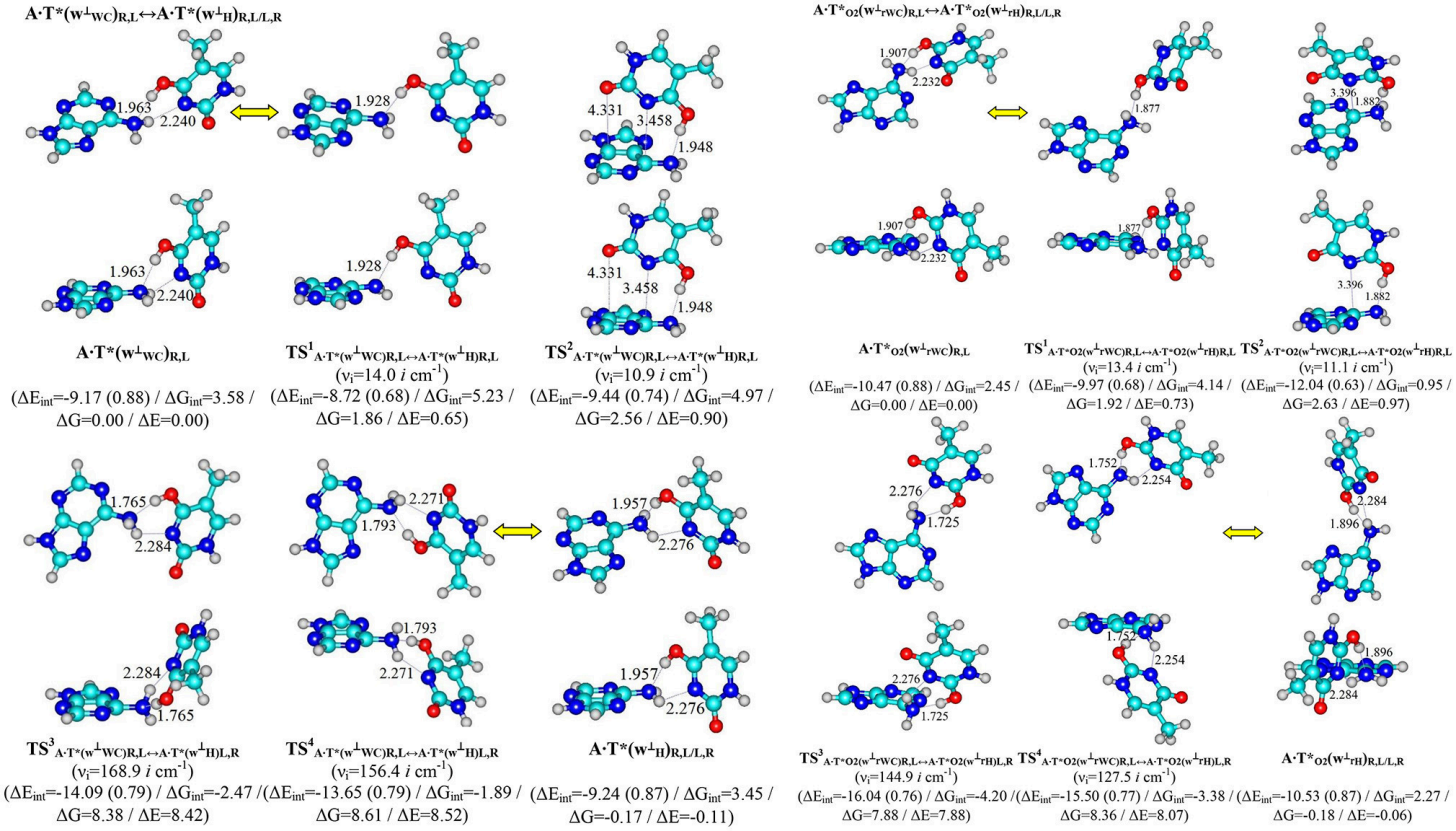
Pathways of the conformational transformations of the complexes - products of the T mutagenic tautomerization in the classical A·T DNA base pairs through the double proton transfer. For the designations see Figure 1.

Performed quantum-chemical calculations completely confirm this assumption (Figures 1, 2 and Supplementary Information, Tables 1, 2).

It was established that novel pathways of the mutagenic tautomerization of the T DNA base in the classical A·T DNA base pairs (Scheme 1) are initiated by their spontaneous conformational transition into the high-energy A·T(w_WC_)_R, L_, A·T(w_H_)_R, L_, A·T(w_rWC_)_R, L_ and A·T(w_rH_)_R, L_ conformers as well as are controlled by the TSs as tight ion pairs (A^+^, protonated by the N6H_2_ amino group)·(T^−^, deprotonated by the N3H imino group) with electronic energy of interaction ΔE_int_ ~145 kcal·mol^−1^. These TSs – ${\text{TS}}^{{\text{A}}^{\text{+}}\cdot {\text{T}}^{-}}{}_{\text{A}\cdot \text{T}({\text{w}}_{\text{WC}})\text{R},\text{L}↔\text{A}\cdot {\text{T}}^{*}({\text{w}}^{⊥}{}_{\text{WC}})\text{L},\text{R}}$ (20.76), ${\text{TS}}^{{\text{A}}^{\text{+}}\cdot {\text{T}}^{-}}{}_{\text{A}\cdot \text{T}({\text{w}}_{\text{rWC}})\text{R},\text{L}↔\text{A}\cdot {\text{T}}^{*}{}_{\text{O}2}({\text{w}}^{⊥}{}_{\text{rWC}})\text{L},\text{R}}$ (23.06), ${\text{TS}}^{{\text{A}}^{\text{+}}\cdot {\text{T}}^{\text{+}}}{}_{\text{A}\cdot \text{T}({\text{w}}_{\text{H}})\text{R},\text{L}↔\text{A}\cdot {\text{T}}^{*}({\text{w}}^{⊥}{}_{\text{H}})\text{L},\text{R}}$ (19.00) and ${\text{TS}}^{{\text{A}}^{+}\cdot {\text{T}}^{-}}{}_{\text{A}\cdot \text{T}({\text{w}}_{\text{rH}})\text{R},\text{L}↔\text{A}\cdot {\text{T}}^{*}{}_{\text{O}2}({\text{w}}^{⊥}{}_{\text{rH}})\text{L},\text{R}}$ (21.48 kcal·mol^−1^) are characterized by the quasi-orthogonal arrangement of the bases relatively each other and are stabilized by the participation of the two non-equivalent strong H-bonds (A)N6^+^H··· N3^−^(T) and (A)N6^+^H··· O4^−^/O2^−^(T) [the first of them is significantly weaker (~15.1–18.6 kcal·mol^−1^), than the second one (~22.5–23.1 kcal·mol^−1^)]. Protonated amino group N6^+^H_3_ of A for these TSs acts simultaneously as donor and acceptor of the H-bonding and has such spatial orientation, that its N6^+^H/N6^+^H′ bond, which is not involved in the H-bonding with T, lies in the plane of the purine ring (Figure 1, Tables 1, 2).

**Table 1:** Energetic characteristics (in kcal·mol^−1^) of the discovered mutagenic tautomerizations of the T DNA base in the classical A·T DNA base pairs *via* the DPT and conformational transformations of their products obtained at the MP2/aug-cc-pVDZ//B3LYP/6-311++G(d,p) level of QM theory in the continuum with ε = 1 at *T* = 298.15 K (see Figures 1, 2).

**Tautomeric / conformational transition**	**ν_i_^a^**	**ΔG^b^**	**ΔE^c^**	***ΔΔ*G_TS_^d^**	***ΔΔ*E_TS_^e^**	***ΔΔ*G^f^**	***ΔΔ*E^g^**
A·T(w_WC_)_R, L_↔A·T*(w^⊥^_WC_)_L, R_	945.3	10.44	9.64	20.76	22.14	10.32	12.49
A·T(w_rWC_)_R, L_↔A·T*_O2_(w^⊥^_rWC_)_L, R_	749.4	14.69	13.66	23.06	23.66	8.37	10.00
A·T(w_H_)_R, L_↔A·T*(w^⊥^_H_)_L, R_	906.9	9.20	8.53	19.00	20.23	9.80	11.70
A·T(w_rH_)_R, L_↔A·T*_O2_(w^⊥^_rH_)_L, R_	704.8	13.75	13.22	21.48	22.41	7.72	9.20
A·T*(w^⊥^_WC_)${}_{\text{R},\text{L}}\overset{1}{↔}$A·T*(w^⊥^_H_)_R, L_	14.0	−0.17	−0.11	1.86	0.65	2.02	0.76
A·T*(w^⊥^_WC_)${}_{\text{R},\text{L}}\overset{2}{↔}$A·T*(w^⊥^_H_)_R, L_	10.9	−0.17	−0.11	2.56	0.90	2.73	1.01
A·T*(w^⊥^_WC_)${}_{\text{R},\text{L}}\overset{3}{↔}$A·T*(w^⊥^_H_)_L, R_	168.9	−0.17	−0.11	8.38	8.42	8.55	8.53
A·T*(w^⊥^_WC_)${}_{\text{R},\text{L}}\overset{4}{↔}$A·T*(w^⊥^_H_)_L, R_	156.4	−0.17	−0.11	8.61	8.52	8.77	8.63
A·T*_O2_(w^⊥^_rWC_)${}_{\text{R},\text{L}}\overset{1}{↔}$A·T*_O2_(w^⊥^_rH_)_R, L_	13.4	−0.18	−0.06	1.92	0.73	2.10	0.79
A·T*_O2_(w^⊥^_rWC_)${}_{\text{R},\text{L}}\overset{2}{↔}$A·T*_O2_(w^⊥^_rH_)_R, L_	11.1	−0.18	−0.06	2.63	0.97	2.81	1.03
A·T*_O2_(w^⊥^_rWC_)${}_{\text{R},\text{L}}\overset{3}{↔}$A·T*_O2_(w^⊥^_rH_)_L, R_	144.9	−0.18	−0.06	7.88	7.88	8.07	7.94
A·T*_O2_(w^⊥^_rWC_)${}_{\text{R},\text{L}}\overset{4}{↔}$A·T*_O2_(w^⊥^_rH_)_L, R_	127.5	−0.18	−0.06	8.36	8.07	8.54	8.13

a *Imaginary frequency at the TS of the tautomeric/conformational transition, cm^−1^*.

b *The Gibbs free energy of the product relatively the reactant of the tautomeric/conformational transition (T = 298.15 K)*.

c *The electronic energy of the product relatively the reactant of the tautomeric/conformational transition*.

d *The Gibbs free energy barrier for the forward tautomeric/conformational transition*.

e *The electronic energy barrier for the forward tautomeric/conformational transition*.

f *The Gibbs free energy barrier for the reverse tautomeric/conformational transition*.

g *The electronic energy barrier for the reverse tautomeric/conformational transition*.

**Table 2:** Electron-topological, geometrical and energetic characteristics of the specific intermolecular contacts – H-bonds and attractive van der Waals contacts in the investigated DNA base pairs and TSs of their tautomeric and conformational transformations obtained at the B3LYP/6-311++G(d,p) level of QM theory in the continuum with ε = 1 at *T* = 298.15 K (see Figures 1, 2).

**Complex**	**AH···*B H*−*bond*/A···B van der Waals contact**	**_ρ_*^a^***	****Δρ**^b^**	***100·ε^c^***	***${\text{d}}_{A\cdot \cdot \cdot B}^{\text{d}}$***	***${\text{d}}_{H\cdot \cdot \cdot B}^{\text{e}}$***	***∠AH*···*B*^f^**	**$E_{AH\cdot \cdot \cdot B}/E_{A\cdot \cdot \cdot B}^{g}$**	**_μ*^h^*_**
A·T(w_WC_)_R, L_	N6H···O4	0.020	0.070	4.53	2.990	2.065	150.0	4.84	2.57
	N3H···N6	0.013	0.040	17.05	3.215	2.345	142.8	2.47
${\text{TS}}^{{\text{A}}^{\text{+}}\cdot {\text{T}}^{-}}{}_{\text{A}\cdot \text{T}({\text{w}}_{\text{WC}})\text{R},\text{L}↔\text{A}\cdot \text{T}*({\text{w}}_{\text{WC}}^{⊥})}$	N6^+^H···O4^−^	0.112	0.077	1.34	2.494	1.379	155.5	23.13**	3.26
	N6^+^H···N3^−^	0.092	0.051	2.61	2.544	1.505	146.4	18.61**
A·T*(w^⊥^_WC_)	O4H···N6	0.028	0.078	6.20	2.929	1.963	166.1	5.56	4.16
	N6H···N3	0.017	0.056	10.90	3.018	2.240	132.0	4.11
A·T(w_rWC_)_R, L_	N6H···O2	0.020	0.071	4.08	2.993	2.062	151.2	3.79	2.68
	N3H···N6	0.011	0.034	20.35	3.273	2.420	141.0	2.11
${\text{TS}}_{\text{A}\cdot \text{T}\left({\text{w}}_{\text{rWC}}\right)R,L↔\text{A}\cdot \text{T}{*}_{\text{O}2}\left({\text{w}}_{\text{rWC}}^{⊥}\right)}^{{\text{A}}^{+}\cdot {\text{T}}^{-}}$	N6^+^H···O4^−^	0.111	0.080	1.70	2.494	1.379	155.7	22.97**	5.14
	N6^+^H···N3^−^	0.078	0.075	2.45	2.573	1.575	144.5	15.44**
A·T*_O2_(${\text{w}}_{\text{rWC}}^{⊥}$)	O2H···N6	0.032	0.084	4.89	2.872	1.907	164.6	5.55	5.56
	N6H···N3	0.018	0.057	10.59	3.006	2.232	131.3	5.44
A·T(w_H_)_R, L_	N6H'···O4	0.018	0.062	4.93	3.010	2.132	143.5	3.70	5.88
	N3H···N6	0.014	0.043	8.80	3.186	2.296	145.1	2.81
${\text{TS}}_{\text{A}\cdot \text{T}\left({\text{w}}_{\text{H}}\right)R,L↔\text{A}\cdot \text{T}*\left({\text{w}}_{\text{H}}^{⊥}\right)}^{{\text{A}}^{+}\cdot \text{T}-}$	N6^+^H'···O4^−^	0.109	0.085	1.39	2.497	1.389	155.2	22.50**	4.54
	N6^+^H'···N3^−^	0.091	0.053	2.62	2.545	1.509	146.1	18.41**
A·T*(w^⊥^_H_)	O4H···N6	0.029	0.079	4.98	2.923	1.957	166.2	5.65	5.23
	N6H'···N3	0.016	0.054	14.79	3.013	2.276	128.1	3.94
A·T(w_rH_)_R, L_	N6H'···O2	0.015	0.052	8.40	3.051	2.213	138.8	3.29	6.10
	N3H···N6	0.016	0.047	6.58	3.155	2.259	145.8	2.98
${\text{TS}}_{\text{A}\cdot \text{T}\left({\text{w}}_{\text{rH}}\right)R,L↔\text{A}\cdot \text{T}{*}_{\text{O}2}\left({\text{w}}_{\text{rH}}^{⊥}\right)}^{{\text{A}}^{+}\cdot {\text{T}}^{-}}$	N6^+^H'···O2^−^	0.109	0.088	1.76	2.497	1.389	155.4	22.50**	5.47
	N6^+^H'···N3^−^	0.076	0.077	2.43	2.576	1.583	144.1	15.07**
A·T*_O2_(w^⊥^_rH_)	O2H···N6	0.033	0.085	3.81	2.863	1.896	164.9	5.77	5.50
	N6H'···N3	0.016	0.053	15.69	3.004	2.284	126.4	5.17
${\text{TS}}^{1}{}_{\text{A}\cdot \text{T}*({\text{w}}_{\text{WC}}^{⊥})\text{R},\text{L}↔\text{A}\cdot \text{T}*({\text{w}}_{\text{H}}^{⊥})\text{R},\text{L}}$	O4H···N6	0.031	0.081	2.57	2.906	1.928	170.0	5.92	4.68
${\text{TS}}^{2}{}_{\text{A}\cdot \text{T}*({\text{w}}_{\text{WC}}^{⊥})\text{R},\text{L}↔\text{A}\cdot \text{T}*({\text{w}}_{\text{H}}^{⊥})\text{R},\text{L}}$	O4H···N6	0.030	0.079	1.84	2.925	1.948	170.0	5.94	4.19
	N3···C6	0.006	0.017	221.70	3.458	-	-	0.84*
	O2···C4	0.002	0.006	121.20	4.331	-	-	0.25*
${\text{TS}}^{3}{}_{\text{A}\cdot \text{T}*({\text{w}}_{\text{WC}}^{⊥})\text{R},\text{L}↔\text{A}\cdot \text{T}*({\text{w}}_{\text{H}}^{⊥})\text{L},\text{R}}$	O4H···N6	0.046	0.097	2.21	2.754	1.765	167.2	8.35	1.19
	N6H'···N3	0.017	0.054	18.48	2.984	2.284	124.3	2.77
${\text{TS}}^{4}{}_{\text{A}\cdot \text{T}*({\text{w}}_{\text{WC}}^{⊥})\text{R},\text{L}↔\text{A}\cdot \text{T}*({\text{w}}_{\text{H}}^{⊥})\text{L},\text{R}}$	O4H···N6	0.043	0.095	2.22	2.775	1.793	165.9	7.99	5.85
	N6H···N3	0.017	0.055	15.55	2.983	2.271	125.3	2.82
${\text{TS}}^{1}{}_{\text{A}\cdot \text{T}{*}_{\text{O}2}({\text{w}}_{\text{rWC}}^{⊥})\text{R},\text{L}↔\text{A}\cdot \text{T}{*}_{\text{O}2}({\text{w}}_{\text{rH}}^{⊥})\text{R},\text{L}}$	O2H···N6	0.035	0.086	2.80	2.854	1.877	168.4	6.73	5.75
${\text{TS}}^{2}{}_{\text{A}\cdot \text{T}{*}_{\text{O}2}({\text{w}}_{\text{rWC}}^{⊥})\text{R},\text{L}↔\text{A}\cdot \text{T}{*}_{\text{O}2}({\text{w}}_{\text{rH}}^{⊥})\text{R},\text{L}}$	O2H···N6	0.035	0.086	2.17	2.863	1.882	170.1	6.80	5.04
	N3···C6	0.005	0.017	205.60	3.396	-	-	0.81*
${\text{TS}}^{3}{}_{\text{A}\cdot \text{T}{*}_{\text{O}2}({\text{w}}_{\text{rWC}}^{⊥})\text{R},\text{L}↔\text{A}\cdot \text{T}{*}_{\text{O}2}({\text{w}}_{\text{rH}}^{⊥})\text{L},\text{R}}$	O2H···N6	0.051	0.097	1.92	2.716	1.725	165.9	9.17	2.73
	N6H'···N3	0.017	0.055	17.37	2.978	2.276	124.3	3.00
${\text{TS}}^{4}{}_{\text{A}\cdot \text{T}{*}_{\text{O}2}({\text{w}}_{\text{rWC}}^{⊥})\text{R},\text{L}↔\text{A}\cdot \text{T}{*}_{\text{O}2}({\text{w}}_{\text{rH}}^{⊥})\text{L},\text{R}}$	O2H···N6	0.048	0.096	1.94	2.735	1.752	164.4	8.81	7.28
	N6H'···N3	0.017	0.057	13.87	2.972	2.254	125.7	3.10

a *The electron density at the (3,−1) BCP of the specific contact, a.u*.

b *The Laplacian of the electron density at the (3, −1) BCP of the specific contact, a.u*.

c *The ellipticity at the (3,−1) BCP of the specific contact*.

*^d^The distance between the A and B atoms of the AH···B / A···B specific contact, Å*.

*^e^The distance between the H and B atoms of the AH···B H-bond, Å*.

f *The H-bond angle, degree*.

*^g^The energy of the specific contact, calculated by Iogansen's (Iogansen, 1999), EML (Espinosa et al., 1998; Mata et al., 2011; marked with an asterisk) or Nikolaienko-Bulavin-Hovorun (Nikolaienko et al., 2012; marked with a double asterisk) formulas, kcal·mol^−1^*.

h *The dipole moment of the complex, D*.

Significantly non-planar A·T^*^(w^⊥^_WC_)_R, L_ (10.44), A·T^*^(w^⊥^_H_)_R, L_ (14.69), A·${\text{T}}_{\text{O}2}^{*}$(w^⊥^_rWC_)_R, L_ (9.20) and A·${\text{T}}_{\text{O}2}^{*}$(w^⊥^_rH_)_R, L_ (13.75) kcal·mol^−1^ complexes (C_1_ symmetry), which are the products of these mutagenic tautomerizations, are stabilized by the two anti-parallel (T)O4H/O2H··· N6(A) (~5.5) and (A)N6H/N6H′··· N3(T) (~4.5 kcal·mol^−1^) H-bonds (Figure 1 and Tables 1, 2).

It is worth to mention that each of the investigated tautomeric and conformational transitions proceed through two mirror-symmetric pathways and do not change *cys/trans* mutual orientation of the N1H and N9H glycosydic bonds of the bases. At the mutagenic tautomeric transformations of the DNA bases some R/L structures transfer into the other L/R structures and *vice versa* (Figures 1, 2).

Terminal tautomerized complexes are conformationally-labile and pairwise interconvert into each other according to four mechanisms (Tables 1, 2).

Two of these tautomerization reactions are controlled by the TSs – ${\text{TS}}^{1,2}{}_{\text{A}\cdot {\text{T}}^{*}({\text{w}}^{⊥}{}_{\text{WC}})\text{R},\text{L}↔\text{A}\cdot {\text{T}}^{*}({\text{w}}_{\text{H}}^{⊥})\text{R},\text{L}}$ (14.0, 10.9 cm^−1^) and ${\text{TS}}^{1,2}{}_{\text{A}\cdot {\text{T}}^{*}{}_{\text{O2}}({\text{w}}^{⊥}{}_{\text{rWC}})\text{R},\text{L}↔\text{A}\cdot {\text{T}}^{*}{}_{\text{O2}}({\text{w}}^{⊥}{}_{\text{rH}})\text{R},\text{L}}$ (13.4, 11.1 cm^−1^) with low values of the imaginary frequencies provided in the brackets. At this, one-single intermolecular (T)O4H/O2H··· N6(A) H-bond between the O4H/O2H hydroxyl groups of T^*^/${\text{T}}_{\text{O}2}^{*}$ and N6 nitrogen atom of the piramidalized amino group of A participates in the stabilization of the TS^1^s. In the case of TS^2^s, when T hangs over A, the (T)O4H/O2H··· N6(A) H-bond coexists together with attractive van der Waals contacts with significantly increased ellipticity – N3··· C6 and O2··· C4 in the case of ${\text{TS}}^{2}{}_{\text{A}\cdot {\text{T}}^{*}({\text{w}}^{⊥}{}_{\text{WC}})↔\text{A}\cdot T^{*}({\text{w}}^{⊥}{}_{\text{H}})}$ and N3··· C6 in the case of ${\text{TS}}^{2}{}_{\text{A}\cdot {\text{T}}^{*}{}_{\text{O}2}({\text{w}}^{⊥}{}_{\text{WC}})↔\text{A}\cdot {\text{T}}^{*}{}_{\text{O}2}({\text{w}}^{⊥}{}_{\text{H}})}$ (Table 2). Notably, conformational transformations, which are controlled by the TS^1^s are the most energetically favorable (1.86 and 1.92) in comparison with the TS^2^s (2.56 and 2.63 kcal·mol^−1^) (Table 1). In these cases R/L structures are converted into the other R/L structures.

Two other mechanisms of the conformational transformations are accompanied by the anisotropic rotation of the A amino group around the exocyclic C6N6 bond, one R/L structures transform into the others L/R structures and *vice versa*. In these cases TSs – ${\text{TS}}^{3,4}{}_{\text{A}\cdot {\text{T}}^{*}({\text{w}}^{⊥}{}_{\text{WC}})\text{R},\text{L}↔\text{A}\cdot {\text{T}}^{*}({\text{w}}^{⊥}{}_{\text{H}})\text{L},\text{R}}$ (8.38 and 8.61 kcal·mol^−1^) and ${\text{TS}}^{3,4}{}_{\text{A}\cdot {\text{T}}^{*}{}_{\text{O}2}({\text{w}}^{⊥}{}_{\text{rWC}})\text{R},\text{L}↔\text{A}\cdot {\text{T}}^{*}{}_{\text{O}2}({\text{w}}^{⊥}{}_{\text{rH}})\text{L},\text{R}}$ (7.88 and 8.36 kcal·mol^−1^) are characterized by the considerably higher values of imaginary frequencies (168.9, 156.4, 144.9, 127.5 cm^−1^) and stabilized by two antiparallel (T)O4/O2H··· N6(A) and (A)N6H/N6H′··· N3(T) H-bonds, the first of which is significantly stronger, then the other one (Table 2).

All tautomeric and conformational transitions without exceptions are dipole-active processes, since they are accompanied by a noticeable change in the dipole moment of the involved complexes (Table 2).

Interestingly, that among all without exception investigated in this work H-bonded structures, the total energy of the intermolecular specific contacts (H-bonds and attractive van der Waals contacts) contribute only a part of the electron energy of the monomer interactions (0.26–0.98; see Figures 1, 2). This result is in a good agreement with the previously published data for the others H-bonded pairs of nucleotide bases (Brovarets' and Hovorun, 2014d).

Notably, the methyl group of the T DNA base does not change its orientation during all, without exception, processes of the tautomeric and conformational transformations. Moreover, the heterocycles of the DNA bases remain planar, despite their ability for the out-of-plane bending (Govorun et al., 1992; Hovorun et al., 1999; Nikolaienko et al., 2011).

Finally, we would like to emphasize the fact that the presence of the conformational transitions between the complexes – products of the A·T^*^(w^⊥^_WC_)R,L↔ A·T^*^(w^⊥^_H_)R,L and A·T^*^_O2_(${\text{w}}_{\text{rWC}}^{⊥}$)R,L↔ A·T^*^_O2_(w^⊥^_rH_)R,L, tautomerizations indicating the close structural relationship between tautomerization the classical A·T(WC) and A·T(H) DNA base pairs, on the one hand, and A·T(rWC) and A·T(rH), on the other hand (Brovarets' et al., 2018b,e).

## Conclusions

In this study, we came out from the existing framework of the mechanisms of the origin of the mutagenic tautomerization of the classical A·T DNA base pairs (Brovarets', 2013b; Brovarets' et al., 2018a,b,c,d,e).

Here we have shed light on the revealed for the first time physico-chemical mechanism of the intrapair mutagenic tautomerization of the T DNA base within the novel highly-energetic conformers of the classical A·T DNA base pairs – Watson-Crick [A·T(w_WC_)], reverse Watson-Crick [A·T(w_rWC_)], Hoogsteen [A·T(w_H_)] and reverse Hoogsteen [A·T(w_rH_)], which have been analyzed in details in our previous paper (Brovarets' et al., 2018a). These reactions – A·T(w_WC_)↔A·T^*^(w^⊥^_WC_), A·T(w_rWC_)↔A·${\text{T}}_{\text{O}2}^{*}$(w^⊥^_rWC_), A·T(w_H_)↔A·T^*^(w^⊥^_H_), A·T(w_rH_)↔A·${\text{T}}_{\text{O}2}^{*}$(w^⊥^_rH_) – proceed through the stepwise proton transfer *via* the TSs as tight A^+^·T^−^ ion pairs, which Gibbs free energy of activation lies in the range of 27.79–29.83 kcal·mol^−1^ at T = 298.15 K, thus creating the substantially non-planar, conformationally-labile complexes – A·T^*^(w^⊥^_WC_), A·${\text{T}}_{\text{O}2}^{*}$(w^⊥^_rWC_), A·T^*^(w^⊥^_H_) and A·${\text{T}}_{\text{O}2}^{*}$(w^⊥^_rH_). Furthermore, formed complexes involving mutagenic T^*^/${\text{T}}_{\text{O}2}^{*}$ tautomers are able to conformationally interconvert between each other according to reaction pathways – A·T^*^(w^⊥^_WC_)↔A·T^*^(w^⊥^_H_) and A·${\text{T}}_{\text{O}2}^{*}$(w^⊥^_rWC_)↔A·${\text{T}}_{\text{O}2}^{*}$(w^⊥^_rH_).

## Author contributions

OB, study conception and design, acquisition of data, drafting of manuscript analysis and interpretation of data, performance of calculations, discussion of the obtained data, preparation of the numerical data for Tables, graphical materials for Figures and text of the manuscript. KT, preparation of the numerical data for Tables and graphical materials for Figures, preparation of the text of the manuscript. AD, analysis and preparation of the current literature survey, discussion of the strategy of the current investigation, analysis of the obtained numerical data, discussion of the obtained data, preparation of the numerical data for Tables, graphical materials for Figures and text of the manuscript. DH, study conception, critical revision of manuscript, proposition of the task of the investigation, discussion of the obtained data, preparation of the text of the manuscript. All authors were involved in the proofreading of the final version of the manuscript.

### Conflict of interest statement

The authors declare that the research was conducted in the absence of any commercial or financial relationships that could be construed as a potential conflict of interest.
