# Supplementary material for: Unexpected Routes of the Mutagenic Tautomerization of the T Nucleobase in the Classical A·T DNA Base Pairs: A QM/QTAIM Comprehensive View
Source: Front Chem. 2018 Nov 27;6:532. doi: 10.3389/fchem.2018.00532 (PMC6277528; doi:10.3389/fchem.2018.00532)
Supplement: Supplementary file 1 [file Data_Sheet_1.PDF]

SUPPORTING INFORMATION

Unexpected routes of the mutagenic tautomerization of the T nucleobase in the classical A·T DNA base pairs: A QM/QTAIM comprehensive view

Ol'ha O. Brovarets'[a,b,✉], Kostiantyn S. Tsiupa[a], Andrii Dinets[c] & Dmytro M. Hovorun[a,d]

[a]Department of Molecular and Quantum Biophysics, Institute of Molecular Biology and Genetics, National Academy of Sciences of Ukraine, 150 Akademika Zabolotnoho Str., 03680 Kyiv, Ukraine

[b]Department of Phramacology, Bogomolets National Medical University, 34 Peremohy Ave., 02000 Kyiv, Ukraine

[c]Department of Surgery #4, Bogomolets National Medical University, 13 Tarasa Shevchenko Blvd., 01601 Kyiv, Ukraine

[d]Department of Pathophysiology, Bogomolets National Medical University, 34 Peremohy Ave., 02000 Kyiv, Ukraine

✉Corresponding author. E-mail: o.o.brovarets@imbg.org.ua

Cartesian coordinates of the investigated complexes:

A·T(w$_{WC}$)

N	-5.043151000000	0.509721000000	0.140018000000
C	-4.818577000000	0.803876000000	-1.189638000000
H	-5.579804000000	1.264076000000	-1.802260000000
N	-3.621838000000	0.470266000000	-1.597443000000
C	-3.018877000000	-0.073935000000	-0.476284000000
C	-1.743051000000	-0.625601000000	-0.250739000000
N	-0.782638000000	-0.659999000000	-1.217659000000
H	-1.088851000000	-0.504312000000	-2.166937000000
H	0.012964000000	-1.281327000000	-1.086975000000
N	-1.454639000000	-1.097583000000	0.971303000000
C	-2.389837000000	-1.018536000000	1.929669000000
H	-2.100733000000	-1.415665000000	2.897887000000
N	-3.624700000000	-0.519964000000	1.846095000000
C	-3.886042000000	-0.062740000000	0.619490000000
H	-5.884750000000	0.674971000000	0.670396000000
N	3.891615000000	1.632318000000	0.577131000000
C	4.589563000000	0.448412000000	0.484003000000
H	5.608576000000	0.485679000000	0.849974000000
C	4.048363000000	-0.679267000000	-0.024398000000
C	4.781872000000	-1.983169000000	-0.133945000000
H	5.800919000000	-1.892635000000	0.247455000000
H	4.266856000000	-2.766916000000	0.427822000000
H	4.826641000000	-2.319367000000	-1.173296000000
C	2.661111000000	-0.622404000000	-0.487870000000
O	2.053630000000	-1.573175000000	-0.963952000000
N	2.037729000000	0.624951000000	-0.361819000000
H	1.059670000000	0.670881000000	-0.641115000000
C	2.572058000000	1.786216000000	0.170708000000
O	1.966809000000	2.832227000000	0.271058000000
H	4.318859000000	2.457102000000	0.970843000000

TS$^{A+·T-}_{A·T(wWC)↔A·T*(w^{⊥}WC)}$

N	-5.027832000000	0.137987000000	-0.144838000000
C	-4.682777000000	0.390701000000	-1.457056000000
H	-5.431180000000	0.618636000000	-2.201888000000
N	-3.396525000000	0.324002000000	-1.680975000000
C	-2.854125000000	0.010409000000	-0.447849000000
C	-1.547368000000	-0.198798000000	-0.002728000000
N	-0.438916000000	-0.104936000000	-0.874320000000
H	-0.729379000000	0.128593000000	-1.821929000000
H	0.339881000000	-0.982077000000	-0.860336000000
N	-1.323200000000	-0.495329000000	1.272738000000
C	-2.375306000000	-0.581374000000	2.103562000000
H	-2.143766000000	-0.823951000000	3.135013000000
N	-3.666218000000	-0.404614000000	1.811651000000
C	-3.854909000000	-0.111561000000	0.529417000000
H	-5.954217000000	0.138514000000	0.254878000000
N	4.031988000000	1.399592000000	0.384561000000
C	4.548152000000	0.138116000000	0.275266000000
H	5.600985000000	0.035000000000	0.512231000000
C	3.782045000000	-0.912170000000	-0.105198000000
C	4.291736000000	-2.317111000000	-0.240340000000
H	5.355368000000	-2.372501000000	0.003614000000
H	3.748120000000	-2.997023000000	0.421426000000
H	4.149789000000	-2.690504000000	-1.258281000000
C	2.388145000000	-0.611267000000	-0.386788000000
O	1.594343000000	-1.544696000000	-0.751000000000
N	1.914405000000	0.651644000000	-0.271418000000
H	0.436100000000	0.563097000000	-0.538081000000
C	2.685940000000	1.712603000000	0.117861000000
O	2.289877000000	2.860992000000	0.236476000000
H	4.609412000000	2.176133000000	0.670860000000

A·T*($w^{\perp}_{WC}$)

N	-5.210910000000	-0.450817000000	-0.191743000000
C	-4.790244000000	-0.794412000000	-1.460465000000
H	-5.474695000000	-1.199910000000	-2.191065000000
N	-3.516550000000	-0.577083000000	-1.660613000000
C	-3.066120000000	-0.060421000000	-0.457773000000
C	-1.807495000000	0.385463000000	-0.020021000000
N	-0.685876000000	0.306055000000	-0.813760000000
H	-0.869190000000	0.220616000000	-1.805610000000
H	0.670235000000	-1.025876000000	-0.322353000000
N	-1.687240000000	0.858681000000	1.224986000000
C	-2.773733000000	0.885477000000	2.012266000000
H	-2.618378000000	1.280397000000	3.011201000000
N	-4.015301000000	0.492490000000	1.721360000000
C	-4.105999000000	0.030185000000	0.474089000000
H	-6.143143000000	-0.525601000000	0.185798000000
N	4.588680000000	1.007774000000	-0.085599000000
C	4.808948000000	-0.286326000000	0.272749000000
H	5.827027000000	-0.546247000000	0.538077000000
C	3.794999000000	-1.190962000000	0.292469000000
C	3.962569000000	-2.633059000000	0.677647000000

H	5.001648000000	-2.843657000000	0.940245000000
H	3.333161000000	-2.890125000000	1.533798000000
H	3.674261000000	-3.298668000000	-0.140535000000
C	2.517068000000	-0.647748000000	-0.086511000000
O	1.494668000000	-1.504759000000	-0.073561000000
N	2.294544000000	0.595329000000	-0.434358000000
H	0.085503000000	0.932290000000	-0.584541000000
C	3.322438000000	1.511167000000	-0.462329000000
O	3.208311000000	2.679129000000	-0.781202000000
H	5.343718000000	1.678642000000	-0.101542000000

A·T(w_{rWC})

N	-5.023908000000	0.920366000000	-0.078218000000
C	-4.815551000000	0.707255000000	-1.425867000000
H	-5.540294000000	1.015814000000	-2.164839000000
N	-3.681079000000	0.110800000000	-1.684945000000
C	-3.104608000000	-0.080811000000	-0.440914000000
C	-1.893031000000	-0.670148000000	-0.030653000000
N	-0.979582000000	-1.150002000000	-0.920242000000
H	-1.294218000000	-1.276624000000	-1.870899000000
H	-0.264318000000	-1.790449000000	-0.584149000000
N	-1.616211000000	-0.720321000000	1.281116000000
C	-2.501097000000	-0.203610000000	2.146764000000
H	-2.224286000000	-0.272909000000	3.194288000000
N	-3.672697000000	0.380150000000	1.887992000000
C	-3.923955000000	0.413464000000	0.577289000000
H	-5.820638000000	1.359811000000	0.356529000000
N	3.783243000000	-1.531757000000	0.114934000000
C	4.645753000000	-0.466261000000	0.281740000000
H	5.655020000000	-0.735003000000	0.568980000000
C	4.263308000000	0.814202000000	0.100161000000
C	5.177225000000	1.990660000000	0.272251000000
H	6.179621000000	1.670398000000	0.563362000000
H	5.248820000000	2.565836000000	-0.654779000000
H	4.792098000000	2.672712000000	1.034892000000
C	2.870368000000	1.072245000000	-0.286730000000
O	2.395220000000	2.174404000000	-0.482783000000
N	2.074873000000	-0.080875000000	-0.428531000000
H	1.098295000000	0.065168000000	-0.674002000000
C	2.455451000000	-1.392443000000	-0.244136000000
O	1.709938000000	-2.349489000000	-0.384608000000
H	4.092993000000	-2.479938000000	0.265567000000

$TS^{A+\cdot T-}_{A\cdot T(wrWC)\leftrightarrow A\cdot T^{*}O2(w^{\perp}rWC)}$

N	5.076252000000	-0.492186000000	-0.167709000000
C	4.742746000000	-0.386100000000	-1.502684000000
H	5.480077000000	-0.525461000000	-2.279711000000
N	3.481418000000	-0.108650000000	-1.705960000000
C	2.944320000000	-0.026475000000	-0.434353000000
C	1.661341000000	0.232820000000	0.048279000000
N	0.574200000000	0.486530000000	-0.825379000000
H	0.861606000000	0.434146000000	-1.801113000000

H	-0.073900000000	1.440311000000	-0.617613000000
N	1.434338000000	0.249251000000	1.355570000000
C	2.463181000000	0.006757000000	2.185052000000
H	2.231237000000	0.027732000000	3.244214000000
N	3.731276000000	-0.256059000000	1.860013000000
C	3.922764000000	-0.262293000000	0.545586000000
H	5.983389000000	-0.702124000000	0.221064000000
N	-3.412089000000	1.682095000000	0.077267000000
C	-4.447673000000	0.781526000000	0.245740000000
H	-5.405465000000	1.215950000000	0.505958000000
C	-4.253909000000	-0.542986000000	0.091101000000
C	-5.340078000000	-1.561767000000	0.261661000000
H	-6.289994000000	-1.090839000000	0.526294000000
H	-5.476154000000	-2.138597000000	-0.657333000000
H	-5.074116000000	-2.281635000000	1.040570000000
C	-2.895577000000	-1.013661000000	-0.264898000000
O	-2.642915000000	-2.202606000000	-0.421540000000
N	-1.911163000000	-0.043352000000	-0.419889000000
H	-0.356513000000	-0.120354000000	-0.657172000000
C	-2.148347000000	1.257222000000	-0.252122000000
O	-1.237854000000	2.144278000000	-0.389011000000
H	-3.546246000000	2.673838000000	0.201681000000

A·T*$_{O2}$(w$^{\perp}_{rWC}$)

N	5.362057000000	0.250106000000	-0.123185000000
C	4.955854000000	1.200133000000	-1.038111000000
H	5.665293000000	1.850821000000	-1.527998000000
N	3.662797000000	1.214301000000	-1.231408000000
C	3.183248000000	0.218134000000	-0.398250000000
C	1.894123000000	-0.277844000000	-0.144339000000
N	0.769654000000	0.270799000000	-0.725791000000
H	0.949128000000	0.804056000000	-1.568020000000
H	-0.377226000000	1.294494000000	0.402501000000
N	1.747017000000	-1.281838000000	0.725399000000
C	2.839145000000	-1.778590000000	1.327208000000
H	2.661617000000	-2.593662000000	2.021411000000
N	4.109514000000	-1.399190000000	1.176649000000
C	4.225549000000	-0.399764000000	0.302445000000
H	6.304139000000	0.054881000000	0.179587000000
N	-3.371566000000	1.388852000000	1.160240000000
C	-4.576716000000	0.774393000000	0.862053000000
H	-5.434442000000	1.136157000000	1.414893000000
C	-4.639178000000	-0.205195000000	-0.060913000000
C	-5.909808000000	-0.908753000000	-0.428522000000
H	-6.758843000000	-0.527473000000	0.143487000000
H	-6.118561000000	-0.789863000000	-1.495110000000
H	-5.816727000000	-1.983557000000	-0.251070000000
C	-3.388720000000	-0.607510000000	-0.749169000000
O	-3.377315000000	-1.481500000000	-1.601118000000
N	-2.209348000000	0.060259000000	-0.385516000000
H	-0.050155000000	-0.334167000000	-0.792356000000
C	-2.243021000000	0.989388000000	0.512702000000

O	-1.154467000000	1.659030000000	0.892892000000
H	-3.303685000000	2.118692000000	1.853134000000

A·T(w_H)

N	-4.081536000000	1.451671000000	1.167644000000
C	-2.952476000000	2.234552000000	1.031815000000
H	-2.885709000000	3.211734000000	1.487249000000
N	-2.015988000000	1.672193000000	0.314794000000
C	-2.551205000000	0.448033000000	-0.048517000000
C	-2.063895000000	-0.625978000000	-0.817589000000
N	-0.815243000000	-0.637579000000	-1.372470000000
H	-0.695188000000	-1.300630000000	-2.125642000000
H	-0.302566000000	0.236698000000	-1.454992000000
N	-2.853617000000	-1.694380000000	-1.003142000000
C	-4.069366000000	-1.707726000000	-0.438648000000
H	-4.660628000000	-2.599669000000	-0.621339000000
N	-4.642513000000	-0.766057000000	0.312606000000
C	-3.840383000000	0.288003000000	0.473420000000
H	-4.926790000000	1.674946000000	1.670162000000
N	4.114777000000	-1.058923000000	0.996162000000
C	4.509910000000	0.199279000000	0.597227000000
H	5.493367000000	0.500933000000	0.937018000000
C	3.733941000000	1.001068000000	-0.162860000000
C	4.138531000000	2.373867000000	-0.612599000000
H	5.131653000000	2.631428000000	-0.239126000000
H	3.426414000000	3.124289000000	-0.259246000000
H	4.147664000000	2.439428000000	-1.703900000000
C	2.422618000000	0.494782000000	-0.573065000000
O	1.632977000000	1.111318000000	-1.275136000000
N	2.108254000000	-0.792765000000	-0.116486000000
H	1.173050000000	-1.132738000000	-0.335948000000
C	2.885186000000	-1.620993000000	0.676222000000
O	2.543282000000	-2.721635000000	1.053578000000
H	4.712562000000	-1.632341000000	1.572046000000

$TS^{A+\cdot T-}_{A\cdot T(wH)\leftrightarrow A\cdot T*(w^{\perp}H)}$

N	3.405805000000	1.688411000000	-1.195435000000
C	2.082433000000	2.062283000000	-1.323975000000
H	1.796435000000	2.924878000000	-1.908044000000
N	1.253675000000	1.282880000000	-0.683693000000
C	2.066187000000	0.331947000000	-0.094020000000
C	1.828808000000	-0.782908000000	0.714314000000
N	0.530461000000	-1.173329000000	1.115595000000
H	0.588824000000	-2.024556000000	1.671657000000
H	-0.166669000000	-0.384758000000	1.620672000000
N	2.850275000000	-1.525606000000	1.135760000000
C	4.095916000000	-1.182270000000	0.766899000000
H	4.892041000000	-1.819554000000	1.136169000000
N	4.460877000000	-0.153799000000	0.001840000000
C	3.419941000000	0.567914000000	-0.401571000000
H	4.212905000000	2.136137000000	-1.602689000000
N	-3.623726000000	-0.530096000000	-1.367159000000

C	-4.079348000000	0.400727000000	-0.474867000000
H	-5.043585000000	0.841473000000	-0.701415000000
C	-3.361390000000	0.744745000000	0.620834000000
C	-3.807744000000	1.761395000000	1.630324000000
H	-4.782731000000	2.175925000000	1.363009000000
H	-3.089378000000	2.582633000000	1.703683000000
H	-3.880745000000	1.319595000000	2.628002000000
C	-2.087369000000	0.064008000000	0.784563000000
O	-1.344346000000	0.332849000000	1.788831000000
N	-1.675023000000	-0.859642000000	-0.115223000000
H	-0.284600000000	-1.263343000000	0.310919000000
C	-2.392416000000	-1.197728000000	-1.229272000000
O	-2.041414000000	-2.011969000000	-2.068809000000
H	-4.162935000000	-0.777878000000	-2.183308000000

A·T*($w^{\perp}_{H}$)

N	3.953486000000	1.157395000000	1.533848000000
C	2.713451000000	1.731830000000	1.730686000000
H	2.555139000000	2.485370000000	2.488236000000
N	1.794986000000	1.272584000000	0.922805000000
C	2.460077000000	0.342103000000	0.143246000000
C	2.054756000000	-0.495666000000	-0.909283000000
N	0.752446000000	-0.560788000000	-1.352465000000
H	0.661307000000	-0.980518000000	-2.269634000000
H	-0.616916000000	-1.413372000000	-0.245082000000
N	2.958950000000	-1.302534000000	-1.478051000000
C	4.220479000000	-1.285540000000	-1.021252000000
H	4.909154000000	-1.960152000000	-1.519863000000
N	4.730579000000	-0.542625000000	-0.038155000000
C	3.809977000000	0.252519000000	0.507499000000
H	4.807434000000	1.355072000000	2.032664000000
N	-4.213307000000	1.156511000000	-0.266724000000
C	-4.494469000000	0.115994000000	0.564006000000
H	-5.472239000000	0.122995000000	1.031256000000
C	-3.586021000000	-0.870284000000	0.784386000000
C	-3.825254000000	-2.047404000000	1.686117000000
H	-4.820502000000	-1.994977000000	2.133057000000
H	-3.088197000000	-2.085070000000	2.492761000000
H	-3.744819000000	-2.989975000000	1.137867000000
C	-2.346386000000	-0.695581000000	0.073589000000
O	-1.427414000000	-1.643384000000	0.266772000000
N	-2.066453000000	0.300377000000	-0.729346000000
H	0.156104000000	0.253603000000	-1.216069000000
C	-2.987531000000	1.297946000000	-0.957125000000
O	-2.815766000000	2.253054000000	-1.690248000000
H	-4.890319000000	1.887731000000	-0.431980000000

A·T(w_{rH})

N	-3.864199000000	1.030584000000	1.630875000000
C	-2.892063000000	1.981892000000	1.393775000000
H	-2.847490000000	2.896936000000	1.966017000000
N	-2.070046000000	1.653921000000	0.431833000000
C	-2.519133000000	0.414700000000	0.008132000000
C	-2.091007000000	-0.464928000000	-1.003713000000
N	-0.982367000000	-0.231735000000	-1.768050000000
H	-0.925018000000	-0.775096000000	-2.617887000000
H	-0.591336000000	0.704733000000	-1.802041000000
N	-2.777070000000	-1.599672000000	-1.202035000000
C	-3.834455000000	-1.861506000000	-0.419954000000
H	-4.347703000000	-2.795928000000	-0.624348000000
N	-4.334708000000	-1.120305000000	0.570214000000
C	-3.641973000000	0.007768000000	0.736888000000
H	-4.605778000000	1.067978000000	2.313228000000
N	3.354107000000	1.572207000000	-0.508322000000
C	4.241251000000	0.867084000000	0.280273000000
H	5.139919000000	1.406567000000	0.553915000000
C	4.009730000000	-0.399596000000	0.681378000000
C	4.954372000000	-1.189940000000	1.537073000000
H	5.841280000000	-0.604417000000	1.787783000000
H	5.270849000000	-2.102797000000	1.025321000000
H	4.467912000000	-1.503897000000	2.464401000000
C	2.756465000000	-1.040730000000	0.261423000000
O	2.419801000000	-2.170447000000	0.562452000000
N	1.927202000000	-0.241976000000	-0.547373000000
H	1.028349000000	-0.637691000000	-0.821062000000
C	2.152682000000	1.054774000000	-0.959690000000
O	1.384371000000	1.691095000000	-1.662042000000
H	3.549998000000	2.520157000000	-0.792117000000

$TS^{A+\cdot T-}_{A\cdot T(wrH)\leftrightarrow A\cdot T^{*}O2(w^{\perp}rH)}$

N	3.431451000000	2.097808000000	-0.394314000000
C	2.131964000000	2.338980000000	-0.795653000000
H	1.830856000000	3.305031000000	-1.173896000000
N	1.344140000000	1.305750000000	-0.668984000000
C	2.159655000000	0.316936000000	-0.151418000000
C	1.958072000000	-1.012119000000	0.225827000000
N	0.697688000000	-1.654785000000	0.125643000000
H	0.771199000000	-2.602350000000	0.492259000000
H	0.141414000000	-1.643504000000	-0.899730000000
N	2.970952000000	-1.730578000000	0.702307000000
C	4.177943000000	-1.150313000000	0.815275000000
H	4.970533000000	-1.777168000000	1.208844000000
N	4.506525000000	0.102511000000	0.501680000000
C	3.474125000000	0.791796000000	0.027150000000
H	4.205321000000	2.745109000000	-0.397392000000
N	-2.974218000000	-0.437692000000	-1.660451000000
C	-3.972972000000	0.211078000000	-0.958682000000
H	-4.841359000000	0.497180000000	-1.540066000000
C	-3.851796000000	0.459481000000	0.360186000000
C	-4.902610000000	1.159222000000	1.168131000000

H	-5.764224000000	1.429526000000	0.552577000000
H	-5.242708000000	0.525154000000	1.991616000000
H	-4.497440000000	2.066166000000	1.625463000000
C	-2.613463000000	0.019326000000	1.042744000000
O	-2.427516000000	0.219973000000	2.237921000000
N	-1.665346000000	-0.639901000000	0.269107000000
H	-0.190437000000	-1.131020000000	0.565424000000
C	-1.823310000000	-0.854260000000	-1.036579000000
O	-0.939544000000	-1.442664000000	-1.748137000000
H	-3.050682000000	-0.615134000000	-2.650167000000

$A{\cdot}T^*_{O2}(w^{\perp}_{rH})$

N	4.050743000000	1.856265000000	0.703194000000
C	2.773891000000	2.378458000000	0.764116000000
H	2.590099000000	3.376447000000	1.134268000000
N	1.855042000000	1.553854000000	0.336532000000
C	2.558295000000	0.420242000000	-0.031610000000
C	2.172776000000	-0.816977000000	-0.571922000000
N	0.854190000000	-1.152576000000	-0.807145000000
H	0.754568000000	-1.930712000000	-1.448685000000
H	-0.295525000000	-1.549239000000	0.646995000000
N	3.113183000000	-1.733334000000	-0.828114000000
C	4.394710000000	-1.438713000000	-0.559374000000
H	5.113664000000	-2.219149000000	-0.787034000000
N	4.890334000000	-0.308573000000	-0.053996000000
C	3.932429000000	0.585697000000	0.189509000000
H	4.911531000000	2.307364000000	0.972975000000
N	-3.187992000000	-0.927870000000	1.541029000000
C	-4.323693000000	-0.256790000000	1.118555000000
H	-5.167547000000	-0.295065000000	1.795855000000
C	-4.340054000000	0.389683000000	-0.063488000000
C	-5.535118000000	1.132446000000	-0.578158000000
H	-6.372377000000	1.078718000000	0.121534000000
H	-5.852010000000	0.726131000000	-1.542427000000
H	-5.286677000000	2.182629000000	-0.753701000000
C	-3.115294000000	0.363656000000	-0.899524000000
O	-3.066508000000	0.923882000000	-1.983061000000
N	-2.007599000000	-0.336795000000	-0.399166000000
H	0.196510000000	-0.396375000000	-0.994750000000
C	-2.081635000000	-0.928116000000	0.747781000000
O	-1.062271000000	-1.611424000000	1.269255000000
H	-3.155168000000	-1.414725000000	2.423766000000

$TS^1_{A{\cdot}T^*(w^{\perp}WC)\leftrightarrow A{\cdot}T^*(w^{\perp}H)}$

N	4.789686000000	0.230406000000	-1.344578000000
C	3.833619000000	-0.101108000000	-2.283502000000
H	4.064025000000	-0.125316000000	-3.338503000000
N	2.665888000000	-0.370945000000	-1.761752000000
C	2.856096000000	-0.210293000000	-0.400257000000
C	2.008479000000	-0.355694000000	0.709561000000
N	0.669892000000	-0.662376000000	0.581835000000
H	0.398751000000	-1.152347000000	-0.262576000000

H	-0.637922000000	0.735373000000	0.348991000000
N	2.500090000000	-0.138814000000	1.933823000000
C	3.788536000000	0.216981000000	2.058052000000
H	4.134324000000	0.379783000000	3.073762000000
N	4.692023000000	0.391101000000	1.092429000000
C	4.175176000000	0.163358000000	-0.115073000000
H	5.754616000000	0.471913000000	-1.511582000000
N	-4.695999000000	-0.906095000000	-0.211106000000
C	-4.825776000000	0.447452000000	-0.268024000000
H	-5.827413000000	0.829501000000	-0.426942000000
C	-3.746046000000	1.260856000000	-0.130185000000
C	-3.811252000000	2.760523000000	-0.181566000000
H	-4.838579000000	3.096805000000	-0.338558000000
H	-3.442656000000	3.205597000000	0.746574000000
H	-3.192802000000	3.156903000000	-0.991466000000
C	-2.505464000000	0.558577000000	0.073630000000
O	-1.421017000000	1.321771000000	0.215907000000
N	-2.367915000000	-0.742266000000	0.128914000000
H	0.246254000000	-1.056737000000	1.413345000000
C	-3.461218000000	-1.566395000000	-0.010371000000
O	-3.430006000000	-2.782240000000	0.027822000000
H	-5.498954000000	-1.510342000000	-0.313414000000

$TS^{2}_{A\cdot T^*(w^{\perp}WC)\leftrightarrow A\cdot T^*(w^{\perp}H)}$

N	-3.031572000000	-1.445966000000	1.118195000000
C	-2.499431000000	-0.806051000000	2.217664000000
H	-2.538803000000	-1.250581000000	3.201304000000
N	-1.969918000000	0.356713000000	1.938216000000
C	-2.148347000000	0.488742000000	0.572235000000
C	-1.769926000000	1.470764000000	-0.345748000000
N	-1.008500000000	2.573567000000	0.033864000000
H	-1.075038000000	3.338669000000	-0.627467000000
H	0.853552000000	2.007991000000	0.117872000000
N	-2.088158000000	1.326783000000	-1.630826000000
C	-2.747691000000	0.214520000000	-2.002788000000
H	-2.987449000000	0.143419000000	-3.058636000000
N	-3.141322000000	-0.802212000000	-1.237973000000
C	-2.810435000000	-0.623389000000	0.039843000000
H	-3.432906000000	-2.370381000000	1.082176000000
N	2.751696000000	-1.948713000000	-0.401169000000
C	3.760013000000	-1.086634000000	-0.104665000000
H	4.759422000000	-1.506262000000	-0.081868000000
C	3.514124000000	0.227595000000	0.144617000000
C	4.600697000000	1.222831000000	0.469912000000
H	5.337225000000	1.291453000000	-0.335991000000
H	4.174990000000	2.214136000000	0.615360000000
H	5.132286000000	0.950475000000	1.386461000000
C	2.123280000000	0.590203000000	0.042325000000
O	1.827037000000	1.879123000000	0.223473000000
N	1.142327000000	-0.241178000000	-0.198273000000
H	-1.153695000000	2.860050000000	0.995288000000
C	1.388672000000	-1.568398000000	-0.442939000000

O	0.541104000000	-2.412676000000	-0.674405000000
H	2.937685000000	-2.924597000000	-0.584017000000

$TS^3_{A \cdot T^*(w^{\perp}WC) \leftrightarrow A \cdot T^*(w^{\perp}H)}$

N	4.803825000000	0.851486000000	-0.457492000000
C	4.261891000000	1.834854000000	0.346068000000
H	4.780447000000	2.766640000000	0.520020000000
N	3.098668000000	1.519516000000	0.849447000000
C	2.842266000000	0.253046000000	0.353381000000
C	1.774515000000	-0.637475000000	0.504077000000
N	0.606876000000	-0.338927000000	1.273592000000
H	0.854267000000	-0.199883000000	2.251335000000
H	-0.890050000000	-1.216327000000	0.948013000000
N	1.829033000000	-1.820159000000	-0.104191000000
C	2.904282000000	-2.118788000000	-0.853395000000
H	2.897473000000	-3.095238000000	-1.325942000000
N	3.975579000000	-1.356232000000	-1.085422000000
C	3.901175000000	-0.185661000000	-0.464837000000
H	5.683824000000	0.877854000000	-0.950069000000
N	-4.024486000000	1.516469000000	-0.635505000000
C	-4.603990000000	0.288182000000	-0.534450000000
H	-5.640655000000	0.217751000000	-0.842665000000
C	-3.904499000000	-0.778984000000	-0.071051000000
C	-4.475672000000	-2.161183000000	0.065771000000
H	-5.519261000000	-2.181960000000	-0.256753000000
H	-3.914742000000	-2.882107000000	-0.535051000000
H	-4.428862000000	-2.508598000000	1.101492000000
C	-2.537022000000	-0.486380000000	0.286849000000
O	-1.828238000000	-1.509766000000	0.740155000000
N	-1.974068000000	0.697948000000	0.193422000000
H	0.172945000000	0.533083000000	0.953970000000
C	-2.682538000000	1.778068000000	-0.276393000000
O	-2.239509000000	2.906433000000	-0.397228000000
H	-4.543845000000	2.310686000000	-0.980842000000

$TS^4_{A \cdot T^*(w^{\perp}WC) \leftrightarrow A \cdot T^*(w^{\perp}H)}$

N	-4.276226000000	1.398071000000	-0.855663000000
C	-3.262958000000	2.284345000000	-0.544509000000
H	-3.343431000000	3.334460000000	-0.786230000000
N	-2.242284000000	1.729263000000	0.049322000000
C	-2.585386000000	0.390559000000	0.144639000000
C	-1.944351000000	-0.735658000000	0.664452000000
N	-0.644630000000	-0.640720000000	1.249996000000
H	-0.693437000000	-0.930913000000	2.225210000000
H	0.530311000000	0.683952000000	0.969049000000
N	-2.559379000000	-1.918146000000	0.604829000000
C	-3.774856000000	-1.995865000000	0.040769000000
H	-4.227663000000	-2.981388000000	0.016628000000
N	-4.489029000000	-1.001081000000	-0.492816000000
C	-3.859540000000	0.163873000000	-0.418411000000
H	-5.151087000000	1.601046000000	-1.314958000000
N	4.319928000000	-0.954186000000	-0.694456000000

C	4.530172000000	0.377204000000	-0.500154000000
H	5.508558000000	0.755368000000	-0.773029000000
C	3.554386000000	1.172342000000	0.008532000000
C	3.712548000000	2.646359000000	0.248396000000
H	4.712911000000	2.979573000000	-0.037612000000
H	2.980230000000	3.221206000000	-0.324943000000
H	3.555015000000	2.895650000000	1.301293000000
C	2.320714000000	0.485257000000	0.307540000000
O	1.348354000000	1.237829000000	0.801931000000
N	2.113758000000	-0.799499000000	0.124552000000
H	0.001393000000	-1.294668000000	0.795376000000
C	3.102867000000	-1.605112000000	-0.388190000000
O	2.997277000000	-2.802000000000	-0.586678000000
H	5.045606000000	-1.546667000000	-1.071539000000

$TS^{1}_{A\cdot T^{*}O2(w^{\perp}rWC)\leftrightarrow A\cdot T^{*}O2(w^{\perp}rH)}$

N	4.955598000000	1.305396000000	0.339660000000
C	3.976119000000	2.278087000000	0.343491000000
H	4.208709000000	3.311453000000	0.555100000000
N	2.784305000000	1.815611000000	0.070861000000
C	2.982196000000	0.460045000000	-0.125752000000
C	2.119456000000	-0.596829000000	-0.454142000000
N	0.754475000000	-0.423383000000	-0.588010000000
H	0.447907000000	0.509629000000	-0.839965000000
H	-0.362427000000	-0.582131000000	0.912307000000
N	2.622197000000	-1.827228000000	-0.591111000000
C	3.939210000000	-2.010360000000	-0.404480000000
H	4.293705000000	-3.028445000000	-0.528651000000
N	4.860580000000	-1.098745000000	-0.090095000000
C	4.330954000000	0.117751000000	0.035271000000
H	5.940886000000	1.427458000000	0.517746000000
N	-3.326204000000	-0.363638000000	1.758267000000
C	-4.575887000000	-0.091239000000	1.227184000000
H	-5.399718000000	-0.127351000000	1.928855000000
C	-4.718166000000	0.195851000000	-0.081620000000
C	-6.040543000000	0.498978000000	-0.717528000000
H	-6.854010000000	0.458195000000	0.010607000000
H	-6.025768000000	1.490700000000	-1.177561000000
H	-6.249794000000	-0.210453000000	-1.522716000000
C	-3.505744000000	0.213576000000	-0.936440000000
O	-3.567957000000	0.467503000000	-2.129459000000
N	-2.277819000000	-0.073596000000	-0.323001000000
H	0.295400000000	-1.136821000000	-1.143032000000
C	-2.237040000000	-0.339295000000	0.940698000000
O	-1.098419000000	-0.621147000000	1.574064000000
H	-3.199131000000	-0.583388000000	2.734381000000

$TS^{2}_{A\cdot T^{*}O2(w^{\perp}rWC)\leftrightarrow A\cdot T^{*}O2(w^{\perp}rH)}$

N	2.497347000000	-1.894746000000	1.189670000000
C	2.187523000000	-1.071667000000	2.250819000000
H	2.106455000000	-1.456537000000	3.256800000000
N	2.015927000000	0.179644000000	1.908553000000

C	2.209854000000	0.182185000000	0.539324000000
C	2.122176000000	1.172387000000	-0.442950000000
N	1.717306000000	2.473770000000	-0.133423000000
H	1.993184000000	2.773384000000	0.795496000000
N	2.364438000000	0.871651000000	-1.715305000000
C	2.664648000000	-0.403059000000	-2.022681000000
H	2.854723000000	-0.601378000000	-3.072208000000
N	2.757306000000	-1.442390000000	-1.196579000000
C	2.512821000000	-1.101607000000	0.066801000000
H	2.611045000000	-2.896777000000	1.205175000000
N	-3.074663000000	1.489168000000	0.462016000000
C	-3.876834000000	0.376705000000	0.282076000000
H	-4.926356000000	0.518525000000	0.509679000000
C	-3.354702000000	-0.790477000000	-0.145155000000
C	-4.184673000000	-2.030002000000	-0.353102000000
H	-3.532951000000	-2.844260000000	-0.668368000000
H	-4.692381000000	-2.336981000000	0.566041000000
H	-4.946648000000	-1.879950000000	-1.123562000000
C	-1.887220000000	-0.856734000000	-0.371395000000
O	-1.321652000000	-1.907973000000	-0.636036000000
N	-1.166786000000	0.341104000000	-0.279200000000
H	1.980073000000	3.136029000000	-0.854997000000
C	-1.750816000000	1.409937000000	0.142246000000
O	-1.112786000000	2.571621000000	0.288200000000
H	-3.450996000000	2.371692000000	0.772568000000
H	-0.153426000000	2.453672000000	0.070050000000

TS$^{3}_{A·T*O2(w^{\perp}rWC)\leftrightarrow A·T*O2(w^{\perp}rH)}$

N	-4.667260000000	1.312494000000	-0.490804000000
C	-4.024736000000	2.154593000000	0.394937000000
H	-4.409743000000	3.140471000000	0.612144000000
N	-2.943188000000	1.641107000000	0.916921000000
C	-2.851264000000	0.383488000000	0.346792000000
C	-1.932245000000	-0.662282000000	0.474261000000
N	-0.767845000000	-0.587661000000	1.301933000000
H	-0.198581000000	0.231110000000	1.060479000000
H	0.540172000000	-1.661113000000	0.966882000000
N	-2.129472000000	-1.784863000000	-0.212747000000
C	-3.202453000000	-1.875820000000	-1.017378000000
H	-3.315963000000	-2.811835000000	-1.553770000000
N	-4.140507000000	-0.951073000000	-1.234895000000
C	-3.925307000000	0.155995000000	-0.535557000000
H	-5.511098000000	1.498451000000	-1.011519000000
N	3.513481000000	-1.606145000000	0.043481000000
C	4.501681000000	-0.724220000000	-0.356014000000
H	5.452370000000	-1.173186000000	-0.615615000000
C	4.260788000000	0.600415000000	-0.406445000000
C	5.286139000000	1.607358000000	-0.830564000000
H	6.235149000000	1.129446000000	-1.085324000000
H	4.931598000000	2.174282000000	-1.695692000000
H	5.459581000000	2.337208000000	-0.035062000000
C	2.911671000000	1.085151000000	-0.026398000000

O	2.629743000000	2.274561000000	-0.054946000000
N	1.967478000000	0.129975000000	0.368441000000
H	-1.033854000000	-0.480429000000	2.278840000000
C	2.282099000000	-1.128801000000	0.388400000000
O	1.434341000000	-2.078907000000	0.750146000000
H	3.670657000000	-2.601735000000	0.078538000000

TS^{4}$_{A·T^*O2(w^{\perp}rWC)\leftrightarrow A·T^*O2(w^{\perp}rH)}$

N	-4.525605000000	1.115098000000	0.948139000000
C	-3.638297000000	2.117110000000	0.606171000000
H	-3.837105000000	3.149967000000	0.853742000000
N	-2.578341000000	1.689127000000	-0.023440000000
C	-2.760108000000	0.318709000000	-0.111333000000
C	-2.006367000000	-0.722340000000	-0.656092000000
N	-0.747906000000	-0.472024000000	-1.286236000000
H	-0.010440000000	-1.043707000000	-0.858070000000
H	0.217915000000	0.971803000000	-1.060633000000
N	-2.470978000000	-1.970094000000	-0.580982000000
C	-3.648360000000	-2.194154000000	0.023493000000
H	-3.977536000000	-3.227166000000	0.059268000000
N	-4.458867000000	-1.292553000000	0.584263000000
C	-3.977855000000	-0.060086000000	0.493099000000
H	-5.402467000000	1.211203000000	1.437528000000
N	3.113099000000	1.767459000000	-0.240864000000
C	4.296214000000	1.214853000000	0.216820000000
H	5.105584000000	1.914652000000	0.384038000000
C	4.397887000000	-0.111741000000	0.429314000000
C	5.648521000000	-0.772505000000	0.923588000000
H	6.448860000000	-0.046046000000	1.083630000000
H	5.457488000000	-1.301761000000	1.861097000000
H	5.991865000000	-1.526414000000	0.209962000000
C	3.211463000000	-0.960299000000	0.161040000000
O	3.239771000000	-2.170005000000	0.333675000000
N	2.051918000000	-0.325077000000	-0.300595000000
H	-0.795305000000	-0.767849000000	-2.259989000000
C	2.039138000000	0.959690000000	-0.479089000000
O	0.976334000000	1.618542000000	-0.915109000000
H	3.015641000000	2.758050000000	-0.403171000000
